# Validation and Psychometric Properties of the Arabic Version of the Duke Anticoagulation Satisfaction Scale (DASS)

**DOI:** 10.3389/fphar.2020.587489

**Published:** 2020-12-17

**Authors:** Maha AlAmmari, Khizra Sultana, Sattam Nawaf AlHarbi, Ashwag Saud Marenga, Abdulrahman AlTuraiki, Abdullah Uthman Althemery, Abdullah Ali Alfaifi, Asma AlShehri, Bander Fahad Aqeel

**Affiliations:** ^1^ Pharmaceutical Care Services, Ministry of National Guard Health Affairs (MNGHA), King Abdullah international Medical Research Centre (KAIMRC), King Saud Bin Abdulaziz University for Health Sciences (KSAU-HS), Riyadh, Saudi Arabia; ^2^ Research Office, King Abdullah International Medical Research Centers (KAIMRC), King Saud Bin Abdulaziz University for Health Sciences (KSAU-HS), Ministry of National Guard Health Affairs (MNGHA), Riyadh, Saudi Arabia; ^3^ Department of Clinical Pharmacy, College of Pharmacy, Prince Sattam bin Abdul Aziz University, Al Kharj, Saudi Arabia

**Keywords:** anticoagulation, quality of life, Duke anticoagulation satisfaction scale, psychometric, warfarin, apixaban, questionnaire, survey

## Abstract

**Background:** To assess the health-related quality of life (HRQoL) of oral anticoagulant therapy users, different types of instruments are available, either general or specific tools like Duke Anticoagulation Satisfaction Scale (DASS). These tools allow the clinician to adjust the treatment regimen to focus on increasing anticoagulation adherence and reduce adverse clinical outcomes. This study aims to validate the translated Arabic version of DASS to assess the satisfaction level of patients using oral anticoagulants in the Arab population.

**Methods:** The Duke Anticoagulation satisfaction scale (DASS) was translated into the Arabic language using MAPI group services. DASS was administered to 505 patients receiving anticoagulation with warfarin or apixaban. The generic scale measuring the quality of life EQ-5D-5L was also administered. Psychometric properties were assessed by Confirmatory Factor Analysis, internal consistency (Cronbach’s Alpha), exploratory factor analysis, convergent and divergent validity, and the correlation between the DASS and demographic variables, clinical characteristics, and the EQ-5D-5L instrument.

**Results:** 439 subjects answered all the questions. From a total of 25 items, 22 grouped into three factors (limitations, positive impact, and negative impact). Each factor had good internal consistency (Cronbach Alpha 0.78–0.88). All the three factors correlated consistently with EQ-5D-5L measuring generic quality of life.

**Conclusion:** The psychometric properties of the Arabic DASS version were comparable to the original English version. The Arabic version of the DASS showed very good reliability and validity. It can be used by health care professionals in other settings of anticoagulation clinics to assess patient’s satisfaction and limitations to anticoagulant treatment.

## Introduction

Anticoagulants are also called blood thinners used to treat and prevent blood clots. Vitamin k antagonist class is the most commonly used, and it is prescribed mostly to patients diagnosed with atrial fibrillation (AF) and to treat different types of venous thromboembolic disorders like pulmonary embolism(PE) and deep vein thrombosis (DVT) (Barrett, Harris et al., Barrett et al., 2014). There are several limitations to the use of vitamin K antagonists (warfarin), which affects the patients’ Health-Related Quality of Life (HRQoL) (Jones et al., 2005; Adam et al., 2012). Firstly, treatment with warfarin requires frequent monitoring to maintain an International Normalized Ratio (INR) within the therapeutic range. 3) Secondly, there are intra-individual and inter-individual differences due to which there is no fixed dose of the drug (Jones et al., 2005). Thirdly, this medication interacts with various foods, herbal medicines, vitamins, and drugs (Nutescu et al., 2011). This interaction may result in an increased INR, resulting in bleeding/bruising or a decreased INR, which may increase harm to the patient. Consequently, this creates problems with the patient’s adherence to the treatment. In addition to the low levels of physical functioning, the disease affects the HRQoL of the patient significantly. In clinical practice, measuring, HRQoL, and satisfaction with warfarin therapy will facilitate interventions to maintain adequate anticoagulation and adherence to treatment. To study the HRQoL of patients on anticoagulant medication, different types of instruments are available. These tools are generic scale (the 36-item Short Form, or the 12-item Short Form) or a condition-specific tool like the Duke Anticoagulation Satisfaction Scale (DASS) developed by Samsa et al. (Samsa et al., 2004) This instrument has been translated and validated till now into Brazilian-Portuguese and Maltase Languages (Pelegrino et al., 2012; Riva et al., 2019). To date, there is no linguistically validated Arabic version of the DASS questionnaire available in the literature. Our study aims to translate and validate the DASS instrument into the Arabic language.

## Materials and Methods

The original DASS included 25 items addressing three dimensions, including the limitations (nine items), “hassles/inconvenience” (eight items), and Psychological impact (five positive items and three negative items). Each question is answered on a 7 point Likert scale with the following options 1) not at all, 2) a little, 3) somewhat, 4) moderately, 5) quite a bit, 6) a lot, and 7) very much (Supplementary *Original DASS Instrument*). If an item is not applied, then the patient is asked to choose the option “not at all.” Six items are reverse coded before the analysis. The final score will range from 25–175, with lower scores indicating higher satisfaction (Samsa et al., 2004). Permission was obtained from the corresponding author of the original DASS publication to use and translate it. The Arabic version of DASS cultural adaptation was done using MAPI group service(Acquadro et al., 1996; Acquadro et al., 2012). MAPI group has been known as the leading authority in linguistic validation and cross-cultural adaptation (Acquadro et al., 2012). It develops scientifically, cross-cultural translation of scientific tools. The cultural adaptation of DASS was performed as follows: 1) Production of the two forward translations into Arabic by two independent translators 2) These translations were analyzed and reconciliation of these translations by the in-country consultant to obtain the first version. 3) Production of the backward translation version 1 into English by one translator. 4) Comparison and analysis of the back translation to the original instrument by the in-country consultant and by the MAPI group. 5) Two proofreading’s of the Arabic version by an independent translator and consultant. 6) Pre-test of the translated version (Supplementary *Arabic Translated DASS-I*).

The EQ-5D-5L incorporates five possible levels (answers) to the five dimensions regarding mobility, self-care, usual activities, pain/discomfort, and anxiety/depression. The EQ-VAS lists the patient’s health using a visual analog scale with two endpoints’ Best imaginable health state’ and “worst imaginable health state.” (EQ-5D-5L, 2015) This instrument has been translated and validated in the Arabic language and is available online to use.

This study received approval by the institutional review board of King Abdullah international medical research Center with protocol no.RC18/123.

### Study Population

In our study, 505 patients completed the Arabic version of the DASS and EQ-5D-5 Lon warfarin and apixaban for various clinical conditions. These patients were enrolled from the anticoagulation clinics and ambulatory care pharmacy at King Abdulaziz Medical City (KAMC), which is a tertiary care center that consists of more than 1,200 beds that covers both adult and pediatric population. KAMC-Riyadh is a referral center for many specialties, including cardiac disease, oncology, and hematology, transplant services, in addition to a lot of different services that serve the National Guard sector and their dependents. The inclusion criteria were: 1) adult patients (age 18 years or older) 2) Use warfarin or apixaban for more than a month and agree to participate in the survey and sign the consent. The excluded patients were those diagnosed with dementia, cognitively impaired, deaf, or mute. The questionnaire was administered in electronic form by the use of i-pads by the pharmacists at King Abdul Aziz Medical City – Riyadh in anticoagulation clinics and ambulatory care pharmacy from January 2019-October 2019. A brief overview of the study and its objectives was given to the participants. After taking their consent, the patients were asked to provide their information on sociodemographic variables (Gender, Age, Nationality, Education, Marital status, Monthly income (SAR), Work status, and Smoking). Clinical characteristics were collected from the hospital BEST CARE electronic system (medication, numbers of years on medication, any hospitalization due to bleeding within the past year, more than one dose change, indication for anticoagulation like stroke, Transient ischemic attack (TIA), Deep Vein thrombosis (DVT), Pulmonary Embolism (PE), Atrial Fibrillation (AF) and Mechanical Valve Replacement (MVR).

### Sample Size

During the validation of the instruments through factor analysis, the general rule of thumb proposed to calculate sample size is a participant: item ratio (N: p). Generally, in psychometric evaluations, a sample size greater than 500 is considered as good, and 1,000 is regarded as excellent (Callum et al., 1999; MacCallum et al., 2001; Boateng et al., 2018). Using large samples decreases the sampling error, which results in stable factor solutions. DASS has 25 items, hence we used the N: p ratio of 20:1, i.e., 20 participants for 1 item. Therefore we calculate the sample size to be 500 participants.

### Statistical Analysis

Statistical analysis was performed using the statistical software SPSS v.26 (SPSS Inc., Chicago, IL, United States), with two- tailed test with *p* < 0.05 considered statistically signiﬁcant. The statistical analysis was performed by assessing any missing data for all variables. Continuous variables were expressed as mean ± SD, and categorical variables were expressed as frequency distributions and percentages. For calculating the DASS score, six items (3h, 4a, 4b, 4f, 4h, and 4j) were reverse-coded according to the original publication (Samsa et al., 2004). The internal consistency (correlation between different items on the same scale or subscale) was assessed using the Cronbach’s alpha coefﬁcient, with a value ≥0.70 considered acceptable (Carmines and Zeller 1979; Webb et al., 2006). First, conﬁrmatory factor analysis (CFA) was performed. The following parameters were calculated: root mean square error of approximation (RMSEA), where values ≤0.05 is a “good ﬁt,” and values ≤0.08 is an “acceptable ﬁt”; standardized root mean squared residual (SRMR), where values ≤0.05 is “good ﬁt,” and values ≤0.10 is an “acceptable ﬁt”; goodness-of ﬁt index (GFI), adjusted goodness-of-ﬁt index (AGFI) and comparative ﬁt index (CFI), where values ≥0.90 are considered “acceptable ﬁt.” (Bentler and Stein 1992; McDonald and Ho 2002; Brown 2006) Convergent and discriminant validity was assessed through factor analysis (Carmines and Zeller 1979; Bhattacherjee 2012). Exploratory factor analysis (EFA) with varimax rotation was performed to examine the latent factor structure (Habing 2003). The known group validity was measured by Pearson’s correlation or point-biserial correlation as appropriate (Cook and Beckman 2006). For this purpose, the various demographic variables were recoded to represent only two groups. Age: ≤50 and ≥51, Education: ≤secondary school and ≥ higher secondary school, Marital status: unmarried and married plus others, Salary: ≤6,000 and ≥6,001, Employment: unemployed and employed. For the EQ-5D-5L, the scales were regrouped into two as “having no problems and slight problems as one category,”, and the rest were combined to represent the second category. The visual analog health status scale was divided as a ≤70 and ≥71%.

## Results

Table 1 describes the demographics, clinical variables, and the response to the Arabic EQ-5D-5L instrument. The minimum age of the participants was 18 years, and the maximum was 89 years. From the 505 participants, 439 were considered for analysis as those with incomplete data were removed. More than half of the participants were females. Greater than 70% of the respondents did not have any problems reporting a good HRQoL.

**TABLE 1:** Describes the demographic clinical characteristics of the subjects.

		Frequency	Percent
Age(SD)	56.33 (15.25)		
Gender			
439	M	201	45.8
	F	238	54.2
Nationality			
439	Saudi	428	97.5
	Non- Saudi	7	1.6
Education			
439	Not Educated	91	20.7
	Primary school	80	18.2
	Secondary school	58	13.2
	High secondary	125	28.5
	Bachelors above	84	19.1
Marital status			
438	Single	34	7.7
	Married	327	74.5
	Divorced	21	4.8
	Widowed	56	12.8
Monthly income (SAR)			
435	Less than 3,500	163	37.1
	3,500–6,000	88	20.0
	6,001–9,000	73	16.6
	9,001–12,000	49	11.2
	More than 12,001	62	14.1
Employment			
439	Employed	69	15.7
	Unemployed	150	34.2
	Retired	136	31.0
	Student	69	15.7
	Housewife	10	2.3
Smoking			
439	Smoker	123	28.0
	Nonsmoker	309	70.4
Medication			
439	Warfarin	361	82.2
	Apixiban	78	17.8
Hospitalized for bleeding last year			
	No	423	96.4
	Yes	15	3.4
Stroke			
439	No	407	93
	Yes	31	7
TIA			
439	No	400	91.1
	Yes	38	8.1
DVT			
439	No	388	88.4
	Yes	50	11.4
AF			
439	No	246	56
	Yes	192	44
MVR			
439	No	239	54.4
	Yes	199	45.3
Mobility			
439	No	349	79.5
	Yes	90	20.5
Self-care			
439	No	401	91.3
	Yes	38	8.7
Usual activities			
439	No	361	82.2
	Yes	78	17.8
Pain/Discomfort			
439	No	385	87.7
	Yes	54	12.3
Anxiety/Depression			
	No	402	91.6
	Yes	37	8.4
Vas analog	≥70 score	335	76.3
436	<70 score	101	23.0

Table 2 describes the answer to each item for the Arabic DASS. The response distribution for all items has a signiﬁcant ﬂoor effect. Except for one item 2c, no signiﬁcant ceiling effect was detected.

**TABLE 2:** Item statistics.

Item	Not at all (1)	A little (2)	Somewhat (3)	Moderately (4)	Quite a bit (5)	A lot (6)	Very much (7)	Mean (SD)Arabic version	Percent (1+2)	Mean (SD) Samsa et al. (2004)
1a	352	43	16	15	6	7	0	1.41 (1.00)	90.0	1.84 (1.37)
1b	388	15	9	10	5	7	5	1.34 (1.09)	91.8	1.36 (0.99)
1c	311	53	25	17	15	12	6	1.71 (1.39)	82.9	1.69 (1.36)
1d	394	20	9	9	2	1	4	1.23 (0.85)	94.3	1.84 (1.78)
1e	344	52	14	16	7	5	1	1.43 (1.01)	90.2	1.88 (1.31)
2a	176	72	40	100	23	13	15	2.59 (1.691)	56.5	2.6 (1.66)
2b	429	2	0	1	1	1	5	1.1 (0.72)	98.2	1.97 (1.89)
2c	76	26	47	46	46	58	140	4.58 (2.54)	23.2	3.02 (2.12)
2d	363	33	22	14	2	3	2	1.35 (0.92)	90.2	2.2 (1.43)
3a	400	15	6	8	3	2	5	1.23 (0.91)	94.5	1.78 (1.22)
3b	349	29	15	18	8	13	7	1.57 (1.36)	86.1	2.09 (1.25)
3c	412	9	10	6	0	1	2	1.13 (0.61)	95.9	1.65 (1.09)
3d	380	35	10	8	5	1	0	1.24 (0.72)	94.5	1.76 (0.97)
3e	393	22	8	6	5	2	3	1.24 (0.85)	94.5	1.76 (1.24)
3f	421	8	1	6	2	1	0	1.09 (0.52)	97.7	1.37 (0.9)
3g	375	32	6	7	9	5	5	1.36 (1.08)	92.7	1.81 (1.17)
3h	287	99	25	11	6	3	8	1.61 (1.16)	87.9	2.9 (2.19)
4a	248	126	41	11	1	7	5	1.71 (1.11)	85.2	2.32 (1.67)
4b	291	102	23	15	2	2	4	1.54 (0.99)	89.5	2.78 (1.66)
4d	236	110	45	26	12	7	3	1.29 (0.87)	78.8	2.55 (1.64)
4f	257	103	25	18	10	14	12	1.86 (1.24)	82	4.15 (2.08)
4g	377	29	13	11	5	3	1	1.89 (1.48)	92.5	2.00 (1.24)
4h	292	96	27	14	6	3	1	1.54 (0.97)	88.4	2.55 (1.6)
4i	345	47	17	17	7	3	3	1.44 (1.05)	89.3	1.75 (1.23)
4j	226	41	34	43	18	14	63	2.73 (2.22)	60.8	2.42 (1.73)

See additional file for item description.

Items 3h, 4a, 4b, 4f and 4j have been reverse coded.

The first 7 columns give the frequencies of each of the 7 response categories (after reverse coding as appropriate) Percent column gives the cumulative percentage of the first two response categories.

The first step was to carry out the confirmatory factor analysis (CFA) in our dataset. The results of the conﬁrmatory factor analysis were unsatisfactory. For the Arabic version of the DASS, RMSEA 0.08 and SRMR 0.073 were above the acceptable value (≤0.08 and ≤0.10, respectively), whereas GFI 0.83, AGFI 0.796, CFI 0.79 and TLI 0.77 were all below the adequate ﬁt level (≥0.90). Since CFA results were unsatisfactory, we explored the item reduction analysis technique using the Classical Test Theory (CTT) (Carmines and Zeller 1979). For the deletion or modification of items, the item-total correlations were estimated. Any item with <0.30 item correlation value was deleted. Hence items 2a, 2b, and 2c were removed. Consequently, the Cronbach alpha was increased from 0.838 to 0.888. EFA was carried out on the resulting 22 items. The five eigenvalues exceeding the unity obtained were 7.32, 2.35, 1.79, 1.12, and 1.04. The last two eigenvalues were close to unity. Hence, only three factors were considered. Thus, rotating factor solutions were fit with two and three factors. The results of the two-factor solution revealed two factors negative and positive, but two items 3a and 4g cross-loaded and 3b and 3c had loading values less than 0.4. Hence we did not further consider this solution. With the three-factor solution, 22 items showed a “simple structure” by having the rotated factors with loadings exceeding 0.4. Six items loaded on the factor positive impact, nine items loaded on the negative impact, and seven items loaded on the factor limitations (Table 3). Therefore, the Arabic language DASS instrument showed a 3-dimensional factor structure with limitations, positive impact, and negative impact subscales.

**TABLE 3:** DASS factor analysis results: 3 –Factor solution.

Item	Negative	Limits	Positive	Item-total correlation	Alpha if item deleted	Communality
1a	—	0.669	—	0.587	0.881	0.567
1b	—	0.799	—	0.468	0.884	0.651
1c	—	0.681	—	0.507	0.883	0.551
1d	—	0.707	—	0.372	0.886	0.515
1e	—	0.722	—	0.666	0.879	0.677
2d	0.584	—	—	0.575	0.882	0.489
3a	0.763	—	—	0.562	0.882	0.628
3b	0.645	—	—	0.423	0.886	0.427
3c	0.596	—	—	0.416	0.886	0.375
3d	0.535	—	—	0.443	0.885	0.403
3e	0.658		—	0.573	0.882	0.553
3f	—	0.403	—	0.416	0.886	0.256
3g	0.573	—	—	0.588	0.881	0.485
3h	—	—	0.698	0.513	0.883	0.544
4a	—	—	0.732	0.425	0.885	0.562
4b	—	—	0.809	0.525	0.883	0.682
4d	—	0.501	—	0.496	0.88	0.407
4f	—	—	0.691	0.585	0.88	0.578
4g	0.703	—	—	0.643	0.881	0.612
4h	—	—	0.738	0.621	0.88	0.649
4i	0.483	—	—	0.492	0.883	0.348
4j	—	—	0.473	0.397	0.883	0.279

Extraction Method: Principal Component Analysis. Rotation Method: Varimax with Kaiser Normalization.

The internal consistency of the Arabic DASS was good with the following Cronbach’s alpha coefﬁcients: *α* = 0.88 for the overall DASS total score; *α* = 0.82 for the limitations subscale (seven items); *α* = 0.78 for the positive (six items) and *α* = 0.84 for the negative (nine items) psychological impact subscales. Table 4 Summarizes the DASS data at the level of the item and reports the distribution of the total score for the overall DASS score and its respective subscales.

**TABLE 4:** Arabic DASS score.

	Minimum	Maximum	Mean(SD)
Negative	8.00	46.00	10.72 (5.36)
Limitations	7.00	38.00	10.06 (5.08)
Positive	6.00	32.00	11.00 (5.73)
Total DASS score	20.1	111.00	31.79 (13.17)

We found a statistically signiﬁcant positive correlation between the DASS total score and its primary subscales. For the Arabic version correlation coefficients between the overall DASS score and the limitations, the subscale is 0.792, the negative impact subscale is 0.851, and the positive impact is 0.799, respectively.

Table 5 describes the convergent and divergent validity. We observed that since the AVE was >0.5 for the subscales, convergent validity is established. The Square root of AVE was higher than the correlation estimate between the subscales. Hence, divergent validity was established.

**TABLE 5:** DASS convergent validity (CV) and divergent validity (DV).

Factors		Factors	Correlation estimate(CE)	Square root of AVE ≥ CE = DV	Average variance extracted(AVE) ≥ 0.5 = CV
Limits	<–>	Negative	0.559	0.831	0.69
Negative	<–>	Positive	0.509	0.813	0.662
Limits	<–>	Positive	0.416	0.85	0.724

Table 6 describes the correlation between the Arabic version of the DASS total score, its subscales, and various demographic characteristics and the co-administered Arabic version of EQ-5D-5L. The overall DASS score, negative impact, limitations, and positive impact subscales consistently correlated with the Arabic version of the EQ-5D-5L.

**TABLE 6:** Pearson’s correlation with Arabic version of the DASS total score and subscales.

	Total	Negative impact	Limitations	Positive impact
Age	**−0.109** ^a^	**−**0.106	**−**0.073	**−0.085** **^a^**
Gender	**0.178** ^**b**^	**0.200** **^b^**	**0.115** **^a^**	**0.118** **^a^**
Nationality	**−**0.004	0.054	0.019	**−**0.077
Education	0.012	0.000	**−**0.017	0.042
Income	**−**0.017	0.004	0.056	**−**0.092
Marital status	**−**0.049	**−**0.092	0.002	**−**0.028
Employment	**−0.231** **^b^**	**−0.245** **^b^**	**−0.161** **^b^**	**−0.157** **^b^**
Years on warfarin	**−**0.043	**−**0.075	**−**0.049	**−**0.017
Years on apixiban	0.197	0.149	**0.271** **^a^**	0.05
Dose change last year	0.055	0.068	0.067	0.004
Hospitalized for bleeding last year	0.033	0.050	0.055	-0.019
Stroke	**−**0.045	**−**0.060	**−**0.035	**−**0.018
TIA	**−**0.032	**−**0.050	**−**0.035	0.004
DVT	**0.235** **^b^**	**0.256** **^b^**	**0.139** **^b^**	**0.171** **^b^**
AF	0.004	**−**0.003	0.025	**−**0.010
MVR	**−0.150** **^b^**	**−0.179** **^b^**	**−0.083**	**−0.100** **^a^**
Smoking	**0.159** **^b^**	**0.138** **^b^**	**0.133** **^b^**	**0.117** **^a^**
EQ-mobility	**−-0.186** **^b^**	**−0.197** **^b^**	**−0.144** **^b^**	**−0.116** **^a^**
EQ-self-care	**−0.221** **^b^**	**−0.235** **^b^**	**−0.168** **^b^**	**−0.138** **^b^**
EQ- usual activities	**−0.281** **^b^**	**−0.302** **^b^**	**−0.199** **^b^**	**−0.186** **^b^**
EQ-pain/discomfort	**−0.305** **^b^**	**−0.264** **^b^**	**−0.230** **^b^**	**−0.249** **^b^**
EQ- anxiety/depression	**−0.354** **^b^**	**−0.313** **^b^**	**−0.243** **^b^**	**−0.305** **^b^**
EQ-VAS	**−0.133** **^b^**	**−0.155** **^b^**	**−**0.083	**−**0.088

a Correlation is significant at the 0.01 level (2-tailed).

b Correlation is significant at the 0.05 level (2-tailed).

NOTE: As lower DASS total scores represent greater satisfaction, a negative correlation means greater satisfaction.

Table 7 reports the statistically significant difference in the mean score for the overall DASS summary score and its subscales between the different groups.

**TABLE 7:** Mean scores between demographics and DASS Arabic version.

		Negative	Limitations	Positive	Total score
		Mean(±SD)	Mean(±SD)	Mean(±SD)	Mean(±SD)
Gender					
	Male	9.52 (3.84)	9.43 (3.82)	10.27 (5.25)	29.23 (10.26)
	Female	11.73 (6.2)	10.6 (5.9)	11.62 (6.04)	33.95 (14.88)
		**t = −4.377; *p* < 0.001**	**t = −2.49; *p* < 0.01**	**t = −2.477; *p* < 0.01**	**t = −3.8; *p* < 0.001**
Employment/retired					
	No	12.35 (6.64)	11.16 (5.7)	12.18 (6.52)	35.71 (15.65)
	Yes	9.71 (4.11)	9.46 (4.6)	10.32 (5.1)	29.50 (10.87)
		**t = 5.11; *p* < 0.001**	**t = 3.397; *p* < 0.001**	**t = 3.3; p < 0.001**	**t = 4.85; p < 0.001**
Age					
	<50	11.61 (6.3)	10.61 (5.86)	11.72 (6.5)	33.95 (15.68)
	>50	10.31 (4.82)	9.81 (4.67)	10.67 (5.32)	30.8 (11.75)
		**t = 2.35; *p* < 0.01**	t = 1.52; *p* = 0.12	t = 1.78; *p* = 0.07	**t = 2.32; p < 0.05**
DVT					
	No	10.2 (4.79)	9.80 (4.88)	10.62 (5.43)	30.63 (12.07)
	Yes	14.6 (7.58)	12.02 (6.1)	13.7 (6.99)	40.32 (17.46)
		**t = −5.64; *p* < 0.001**	**t= −2.92; *p* = 0.004**	**t = −3.62; *p* < 0.001**	**t = −5.03; *p* < 0.001**
MVR					
	No	11.56 (6.35)	10.44 (6.02)	11.5 (5.95)	33.51 (15.07)
	Yes	9.67 (3.59)	9.59 (3.622)	10.35 (5.34)	29.61 (9.98)
		**t = 3.73; *p* < 0.001**	t = 1.74; *p* = 0.082	**t = 2.10; *p* < 0.05**	**t = 3.12; *p* = 0.001**

NOTE: lower mean DASS scores represent greater satisfaction.

## Discussion

In our study, for the ﬁrst time, the anticoagulation satisfaction scale DASS was translated into the Arabic language. The questionnaire was administered to a group of patients on warfarin and apixaban treatment for different clinical conditions. We assessed the psychometric properties and the validity of the Arabic translation. The Arabic version of DASS showed excellent reliability and validity.

Concerning the response to items, we observed that the answer to one item “2c” about limitation of over the counter drugs showed a significant ceiling effect. This effect was similar to what was reported in the previous studies, which suggests the intrinsic character of the questionnaire (Samsa et al., 2004; Riva et al., 2019).

The results of CFA in our study did not confirm the factor structure suggested by Samsa et al. Similarly, other studies in the literature were not able to confirm similar domains suggested by Samsa et al. except the Turkish version of DASS (Samsa et al., 2004; Gafou et al., 2007; Pelegrino et al., 2012; Yıldırım and Temel 2014; Riva et al., 2019). The RMSEA value for our data was 0.08, and SRMR was close to 0.05, which showed an acceptable fit compared to the Maltese version (Riva et al., 2019). The GFI, AGFI, CFI, and TLI were also very close to 0.9 in Arabic versions as compared to the Maltese version, where they were below 0.9 value (Riva et al., 2019). This indicates that the factor structure suggested by Samsa et al. performed better in our study compared to other studies. This difference can be attributed to the different types of health systems and cultural differences in different countries.

The overall reliability for the Arabic translation was found to be very good, with alpha 0.88 similar to previous studies (Samsa et al., 2004; Gafou et al., 2007; Riva et al., 2019). However, the Turkish version had the highest Alpha of 0.91(Yıldırım and Temel 2014). In our research, the EFA revealed only three dimensions that were divided into negative impact, positive impact, and limitations. The original, Maltese, Portuguese, and Turkish version DASS reported four subscales as limitations, hassles and burdens, and psychological impact which were divided as negative and positive impact. In our study, the items about burden were distributed between the limitations and negative impact. We did not get all items of hassles and burdens into one factor, and psychological impact did not divide into negative and positive like other studies (Samsa et al., 2004; Yıldırım and Temel 2014; Riva et al., 2019). The value in the Maltese version for the positive and negative impact subscale (*α* = 0.65 and *α* = 0.64) and Portuguese versions (α = 0.67 and *α* = 0.38) was lower compared to our positive and negative subscale respectively (*α* = 0.78 and *α* = 0.84) (Pelegrino et al., 2012; Riva et al., 2019). The Greek version had only two subscales positive impact, and negative (*α* = 0.78 and *α* = 0.88) had values similar to our subscales (Gafou et al., 2007). The Portuguese subscale limitations with nine items and an Arabic subscale with seven items had similar reliability *α* = 0.72 and *α* = 0.78, respectively. The Maltese limitations scale with nine items and the original version DASS subscale limitation with ten items had similar reliability *α* = 0.86 and *α* = 0.87, respectively.

On assessing the construct validity, a signiﬁcant positive correlation was found between the overall DASS score and its subscales, in line with the fact that the DASS total score is the sum of it’s three subscales (limitations, positive and negative impact). The average variance extracted (AVE) should be at least 0.5 or more to establish convergent validity (Cook and Beckman 2006). For all the three, the subscale’s AVE was greater than 0.6; hence convergent validity was established. The square root of the AVE, known as the divergent value (DV), should be higher than the correlation estimate between the scales (Cook and Beckman 2006). In our study, the DV was at least 0.8, and the CE was 0.5 and below. Therefore, we established divergent validity of the scales. None of the previous studies have reported this type of validity for the DASS scale.

For the known-group validity, the Arabic version of the DASS correlated with gender, similar to previous studies in the literature (Samsa et al., 2004; Riva et al., 2019). It was observed that the males had greater satisfaction with their anticoagulation treatment compared to females and education did not correlate significantly with overall DASS score and its subscale. Satisfaction with anticoagulation and being a male in our study is similar to a survey by Balkhi who reported that male participants and those with higher secondary education were more satisfied with their anticoagulation therapy (Balkhi et al., 2018). Our findings are similar to the Turkish study which reported no relation between overall DASS score and its subscales with education (Yıldırım and Temel 2014). We found that with employment and increasing age, there was increased satisfaction with anticoagulation. This is in line with previous studies that reported lower satisfaction with younger age but higher satisfaction with being employed and retired (Samsa et al., 2004; Pelegrino et al., 2012; Radaideh and Matalqah 2018).

In patients diagnosed with MVR, AF, and DVT, anticoagulation is the primary treatment to prevent stroke and recurrent VTE (Jones et al., 2005; Kearon et al., 2016; National Health Service, 2017; Nishimura et al., 2017). We found that patients who suffered from DVT had lower satisfaction compared to those who did not have DVT. The patients who had MVR were more satisfied with their anticoagulation compared to those who did not have MVR. Further evaluation of the risk factors related to the difference in the satisfaction levels with anticoagulation in DVT and MVR patients’ needs to be performed in future research. Overall it was observed that since the patients did not have any hospitalization in the previous year due to anticoagulation related bleeding and more than 75% of patients on the VAS analog reported greater than 70% hence they reported good perceived HRQoL.

The DASS was initially developed in English, and, so far, it has been translated and validated in Greek, Brazilian-Portuguese, Malay, Turkish, and Maltese languages. In our study, it was translated in Arabic and tested in 439 patients, which is the largest sample population as compared with the previous studies (Turkish -229, Malay-339, Greek 77 patients, Brazilian-Portuguese-180). We carried out a robust analysis to establish the reliability and validity of the Arabic version. In our population, we did not have the right amount of bilingual people; hence the original English version of the instrument was not evaluated. We were not able to collect data on patients’ INR and bleeding events. Therefore, we were not able to establish, if most of the patients adhered to their therapy though they reported satisfaction with their treatment. In our study, patients were enrolled from a large government facility in Saudi Arabia. These results cannot be generalized to all anticoagulated patients in the Arab world. More studies need to be conducted in other types of health care systems from different regions in the Arab world to assess the validity and reliability of the Arabic version of DASS.

## Conclusion

The Arabic version of DASS showed excellent reliability and validity. These ﬁndings were comparable to the original English version. The results of our study suggest that the Arabic version of DASS is a valid and reliable instrument to assess the level of satisfaction of Arabic speaking anticoagulated patients. It can be used by health care professionals working in other settings of anticoagulation clinics for future research studies assessing patients’ satisfaction and limitations to anticoagulant treatment.

## Data Availability Statement

The raw data supporting the conclusions of this article will be made available by the authors, without undue reservation.

## Ethics Statement

The studies involving human participants were reviewed and approved by Institutional Review Board, King Abdullah International Medical Research Centre. The patients/participants provided their written informed consent to participate in this study.

## Author Contributions

MA, KS, and AAT conceived of the study, proposal development, design, coordination, and acquisition of data. MA participated in writing the manuscript. KS participated in data analysis, data interpretation, writing, and critically revising the manuscript. SA, AM, AAT, AUA, AAA, AAF, AAS, and BF participated in data collection. MA and KS contributed equally to the manuscript. All authors read and approved the final manuscript.

## Conflict of Interest

The authors declare that the research was conducted in the absence of any commercial or financial relationships that could be construed as a potential conflict of interest.
