# Supplementary material for: Validation and Psychometric Properties of the Arabic Version of the Duke Anticoagulation Satisfaction Scale (DASS)
Source: Front Pharmacol. 2020 Dec 17;11:587489. doi: 10.3389/fphar.2020.587489 (PMC7773898; doi:10.3389/fphar.2020.587489)
Supplement: Supplementary file 1 [file datasheet1.pdf]

مقياس رضا المرضى عن تناول علاج تخثر الدم

نود التعرف على تأثير علاج منع تخثر الدم عليك، وما الذي تعرفه وتشعر به حيال علاجك بمضاد تخثر الدم. يُرجى وضع علامة على الإجابة التي تطابق وضعك بأفضل صورة. إذا كان أحد الأسئلة لا ينطبق عليك، فضع علامة على إجابة "لا على الإطلاق".

في جميع الأسئلة يوجد 7 خيارات للإجابة.

☐ لا على الإطلاق ☐ قليلًا ☐ إلى حدٍ ما ☐ بشكل متوسط ☐ إلى حد كبير ☐ كثيرًا ☐ كثيرًا جدًا

عندما تتناول علاج منع تخثر الدم، تصبح أكثر عرضة للنزف أو الإصابة بالكدمات. ربما تقل أنشطتك نتيجة لذلك. تقلل تعني أنك تؤدي النشاط بمستوى أقل، أو لم تعد تؤدي النشاط على الإطلاق.

1. إلى أي مدى يجعلك احتمال إصابتك بالنزيف أو الكدمات تقلل مشاركتك في الأنشطة البدنية (على سبيل المثال الأعمال المنزلية أو تنسيق الحديقة أو المشي أو ممارسة الرياضة أو أي شيء آخر تقوم به عادة)؟

☐ لا على الإطلاق ☐ قليلًا ☐ إلى حدٍ ما ☐ بشكل متوسط ☐ إلى حد كبير ☐ كثيرًا ☐ كثيرًا جدًا

2. إلى أي مدى يجعلك احتمال إصابتك بالنزيف أو الكدمات تقلل من السفر؟

☐ لا على الإطلاق ☐ قليلًا ☐ إلى حدٍ ما ☐ بشكل متوسط ☐ إلى حد كبير ☐ كثيرًا ☐ كثيرًا جدًا

3. إلى أي مدى يجعلك احتمال إصابتك بالنزيف أو الكدمات تقلل من الحصول على الرعاية الطبية التي تحتاج إليها (على سبيل المثال زيارة طبيب الأسنان أو أخصائي المعالجة اليدوية أو الطبيب الذي تختاره)؟

☐ لا على الإطلاق ☐ قليلًا ☐ إلى حدٍ ما ☐ بشكل متوسط ☐ إلى حد كبير ☐ كثيرًا ☐ كثيرًا جدًا

4. إلى أي مدى يقلل احتمال إصابتك بالنزيف أو الكدمات من قدرتك على العمل مقابل أجر؟

☐ لا على الإطلاق ☐ قليلًا ☐ إلى حدٍ ما ☐ بشكل متوسط ☐ إلى حد كبير ☐ كثيرًا ☐ كثيرًا جدًا

5. بشكل عام، إلى أي مدى يؤثر احتمال إصابتك بالنزيف أو الكدمات على حياتك اليومية؟

☐ لا على الإطلاق ☐ قليلًا ☐ إلى حدٍ ما ☐ بشكل متوسط ☐ إلى حد كبير ☐ كثيرًا ☐ كثيرًا جدًا

 حقوق النشر Greg Samsa, Duke University ,2004

قد يعني تناولك لعلاج منع تخثر الدم أن تقوم بتغيير بعض عاداتك الأخرى أيضًا.

6. إلى أي مدى يجعلك تناول علاج منع تخثر الدم تقلل من اختيارك للطعام (النظام الغذائي)؟

☐ لا على الإطلاق ☐ قليلًا ☐ إلى حدٍ ما ☐ بشكل متوسط ☐ إلى حد كبير ☐ كثيرًا ☐ كثيرًا جدًا

7. إلى أي مدى يجعلك تناول علاج منع تخثر الدم تقلل من المشروبات الكحولية التي ربما تود أن تشربها؟

☐ لا على الإطلاق ☐ قليلًا ☐ إلى حدٍ ما ☐ بشكل متوسط ☐ إلى حد كبير ☐ كثيرًا ☐ كثيرًا جدًا

8. إلى أي مدى يجعلك تناول علاج منع تخثر الدم تقلل من الأدوية التي تُصرف دون وصفة طبية (على سبيل المثال الأسبرين وبانادول والفيتامينات) التي ربما تود أن تتناولها؟

☐ لا على الإطلاق ☐ قليلًا ☐ إلى حدٍ ما ☐ بشكل متوسط ☐ إلى حد كبير ☐ كثيرًا ☐ كثيرًا جدًا

9. بشكل عام، إلى أي مدى يؤثر تناول علاج منع تخثر الدم على حياتك اليومية؟

☐ لا على الإطلاق ☐ قليلًا ☐ إلى حدٍ ما ☐ بشكل متوسط ☐ إلى حد كبير ☐ كثيرًا ☐ كثيرًا جدًا

 حقوق النشر 2004, Greg Samsa, Duke University

تناولك لعلاج منع تخثر الدم يعني قيامك بعمل الكثير من الأشياء، بعضها بصفة يومية وبعضها بوتيرة أقل حدوثًا.
الواجبات اليومية قد تشمل: تذكرك لتناول علاجك في وقت معين، وتناول الجرعة الصحيحة من الدواء، وعدم الإفراط في شرب الكحوليات، وإتباع نظام غذائي معتدل وتجنب الإصابة بالكدمات والنزيف وهلم جرا.
الواجبات المتقطعة قد تشمل: الانتقال إلى العيادة لإجراء فحص للدم، والاتصال بالعيادة في حالة حدوث نزيف أو أحداث أخرى هامة، وهلم جرا.

10. ما مدى الضيق (الانزعاج) من الواجبات اليومية لعلاج منع تخثر الدم؟

☐	☐	☐	☐	☐	☐	☐
لا على الإطلاق	قليلًا	إلى حدٍ ما	بشكل متوسط	إلى حد كبير	كثيرًا	كثيرًا جدًا

11. ما مدى الضيق (الانزعاج) من الواجبات المتقطعة لعلاج منع تخثر الدم؟

☐	☐	☐	☐	☐	☐	☐
لا على الإطلاق	قليلًا	إلى حدٍ ما	بشكل متوسط	إلى حد كبير	كثيرًا	كثيرًا جدًا

بالنظر إلى علاج منع تخثر الدم بصورة عامة (أي كل من الواجبات اليومية والمتقطعة)، فضلًا أجب عن الأسئلة التالية.

12. ما مدى الالتباس الذي تجده في تناولك لعلاج منع تخثر الدم؟

☐	☐	☐	☐	☐	☐	☐
لا على الإطلاق	قليلًا	إلى حدٍ ما	بشكل متوسط	إلى حد كبير	كثيرًا	كثيرًا جدًا

13. ما مدى قضاء الوقت الذي تجده يمضي أثناء تناولك لعلاج منع تخثر الدم؟

☐	☐	☐	☐	☐	☐	☐
لا على الإطلاق	قليلًا	إلى حدٍ ما	بشكل متوسط	إلى حد كبير	كثيرًا	كثيرًا جدًا

14. ما مدى الإحباط الذي تجده نتيجة لتناولك علاج منع تخثر الدم؟

☐	☐	☐	☐	☐	☐	☐
لا على الإطلاق	قليلًا	إلى حدٍ ما	بشكل متوسط	إلى حد كبير	كثيرًا	كثيرًا جدًا

15. ما مدى الألم الذي تجده نتيجة لتناولك علاج منع تخثر الدم؟

☐	☐	☐	☐	☐	☐	☐
لا على الإطلاق	قليلًا	إلى حدٍ ما	بشكل متوسط	إلى حد كبير	كثيرًا	كثيرًا جدًا

16. بصفة عامة، ما مدى العبء الذي تجده نتيجة لتناولك علاج منع تخثر الدم؟

☐	☐	☐	☐	☐	☐	☐
لا على الإطلاق	قليلًا	إلى حدٍ ما	بشكل متوسط	إلى حد كبير	كثيرًا	كثيرًا جدًا

17. بصفة عامة، ما مدى الثقة التي تشعر بها في إدارتك لعلاج منع تخثر الدم؟

☐	☐	☐	☐	☐	☐	☐
لا على الإطلاق	قليلًا	إلى حدٍ ما	بشكل متوسط	إلى حد كبير	كثيرًا	كثيرًا جدًا

حقوق النشر 2004, Greg Samsa, Duke University

الأسئلة الأخيرة تدور حول معرفتك وشعورك حيال علاجك لمنع تخثر الدم.

18. ما مدى شعورك بالارتياح مع إدراك السبب الطبي وراء علاجك لمنع تخثر الدم؟

☐	☐	☐	☐	☐	☐	☐
لا على الإطلاق	قليلًا	إلى حدٍ ما	بشكل متوسط	إلى حد كبير	كثيرًا	كثيرًا جدًا

19. ما مدى شعورك بالطمأنينة بسبب علاجك لمنع تخثر الدم؟

☐	☐	☐	☐	☐	☐	☐
لا على الإطلاق	قليلًا	إلى حدٍ ما	بشكل متوسط	إلى حد كبير	كثيرًا	كثيرًا جدًا

20. بصفة عامة، ما مدى الأثر السلبي الذي سببه علاج منع تخثر الدم على حياتك؟

☐	☐	☐	☐	☐	☐	☐
لا على الإطلاق	قليلًا	إلى حدٍ ما	بشكل متوسط	إلى حد كبير	كثيرًا	كثيرًا جدًا

21. ما مدى شعورك بالقلق حيال النزيف والكدمات؟

☐	☐	☐	☐	☐	☐	☐
لا على الإطلاق	قليلًا	إلى حدٍ ما	بشكل متوسط	إلى حد كبير	كثيرًا	كثيرًا جدًا

22. بصفة عامة، ما مدى الأثر الإيجابي الذي سببه علاج منع تخثر الدم على حياتك؟

☐	☐	☐	☐	☐	☐	☐
لا على الإطلاق	قليلًا	إلى حدٍ ما	بشكل متوسط	إلى حد كبير	كثيرًا	كثيرًا جدًا

23. بصفة عامة، ما مدى شعورك بالرضا عن علاج منع تخثر الدم؟

☐	☐	☐	☐	☐	☐	☐
لا على الإطلاق	قليلًا	إلى حدٍ ما	بشكل متوسط	إلى حد كبير	كثيرًا	كثيرًا جدًا

24. مقارنة بأنواع العلاج الأخرى التي تناولتها، ما مدى صعوبة إدارة علاج منع تخثر الدم؟

☐	☐	☐	☐	☐	☐	☐
لا على الإطلاق	قليلًا	إلى حدٍ ما	بشكل متوسط	إلى حد كبير	كثيرًا	كثيرًا جدًا

25. ما مدى احتمال أن توصي شخصًا آخر مصابًا بمرضك أو يعاني من نفس حالتك الطبية بأن يتناول هذا النوع من علاج منع تخثر الدم؟

☐	☐	☐	☐	☐	☐	☐
لا على الإطلاق	قليلًا	إلى حدٍ ما	بشكل متوسط	إلى حد كبير	كثيرًا	كثيرًا جدًا

حقوق النشر Greg Samsa, Duke University ,2004
