# Supplementary material for: Validation and Psychometric Properties of the Arabic Version of the Duke Anticoagulation Satisfaction Scale (DASS)
Source: Front Pharmacol. 2020 Dec 17;11:587489. doi: 10.3389/fphar.2020.587489 (PMC7773898; doi:10.3389/fphar.2020.587489)
Supplement: Supplementary file 2 [file datasheet2.doc]

**DASS instrument**

We would like to know how your anti-clot treatment (warfarin/coumadin) affects you, and what you know and feel about your anti-clot treatment. Please check the answer that best fits your situation. If a question does not apply to you, then check “not at all”.

When you have anti-clot treatment you tend to bleed or bruise more easily. You may limit your activities as a result. Limit means you do less of the activity, or no longer perform the activity at all.

**1a. How much does the possibility of bleeding or bruising limit you from taking part in physical activities (for example, housework, gardening, dancing, sports, or anything else you would usually do)?**

1. not at all,(2) a little),(3) somewhat,(4) moderatley,(5) quite a bit),(6) a lot,(7) very much

**1b. how much does the possibility of bleeding or bruising limit you from traveling?**

1. not at all,(2) a little),(3) somewhat,(4) moderatley,(5) quite a bit),(6) a lot,(7) very much

**1c. how much does the possibility of bleeding or bruising limit you from getting the medical care you need (for example, visiting a dentist, chiropractor, or doctor of your choice)?**

1. not at all,(2) a little),(3) somewhat,(4) moderatley,(5) quite a bit),(6) a lot,(7) very much

**1d. How much does the possibility of bleeding or bruising limit your ability to work for pay?**

1. not at all,(2) a little),(3) somewhat,(4) moderatley,(5) quite a bit),(6) a lot,(7) very much

**1e. Overall, how much does the possibility of bleeding or bruising affect your daily life?**

(1) not at all,(2) a little),(3) somewhat,(4) moderatley,(5) quite a bit),(6) a lot,(7) very much

Being on anti-clot treatment may mean changing some of your other habits as well.

**2a. How much does anti-clot treatment limit your choice of food (diet)?**

1. not at all,(2) a little),(3) somewhat,(4) moderatley,(5) quite a bit),(6) a lot,(7) very much

**2b. How much does anti-clot treatment limit the alcoholic beverages you might wish to drink?**

1. not at all,(2) a little),(3) somewhat,(4) moderatley,(5) quite a bit),(6) a lot,(7) very much

**2c. How much does anti-clot treatment limit the over-the-counter medications (for example, aspirin, ibuprofen, vitamins) you might wish to take?**

1. not at all,(2) a little),(3) somewhat,(4) moderatley,(5) quite a bit),(6) a lot,(7) very much

**2d. Overall, how much does anti-clot treatment affect your daily life?**

(1) not at all,(2) a little),(3) somewhat,(4) moderatley,(5) quite a bit),(6) a lot,(7) very much

Being on anti-clot treatment means doing a lot of things, some every day and some less often.

Daily tasks could include: remembering to take your medicine at a certain time, taking the correct does of your medicine, not drinking much alcohol, following a moderate diet, avoiding bruising and bleeding, and so forth.

Occasional tasks could include: traveling to the clinic for blood check-ups, contacting the clinic in case of bleeding or other important events, and so forth.

**3a. How much of a hassle (inconvenience) are the daily tasks of anti-clot treatment?**

1. not at all,(2) a little),(3) somewhat,(4) moderatley,(5) quite a bit),(6) a lot,(7) very much

**3b. How much of a hassle (inconvenience) are the occasional tasks of anti-clot treatment?**

(1) not at all,(2) a little),(3) somewhat,(4) moderatley,(5) quite a bit),(6) a lot,(7) very much

Considering anti-clot treatment as a whole (that is, both the daily and occasional tasks), please consider the following.

**3c. How complicated do you find your anti-clot treatment to be?**

1. not at all,(2) a little),(3) somewhat,(4) moderatley,(5) quite a bit),(6) a lot,(7) very much

**3d. How time-consuming do you find your anti-clot treatment to be?**

1. not at all,(2) a little),(3) somewhat,(4) moderatley,(5) quite a bit),(6) a lot,(7) very much

**3e. How frustrating do you find your anti-clot treatment to be?**

1. not at all,(2) a little),(3) somewhat,(4) moderatley,(5) quite a bit),(6) a lot,(7) very much

**3f. How painful do you find your anti-clot treatment to be?**

1. not at all,(2) a little),(3) somewhat,(4) moderatley,(5) quite a bit),(6) a lot,(7) very much

**3g. Overall, how much of a burden do you find your anti-clot treatment to be?**

1. not at all,(2) a little),(3) somewhat,(4) moderatley,(5) quite a bit),(6) a lot,(7) very much

**3h. Overall, how confident are you about handling your anti-clot treatment**

(1) not at all,(2) a little),(3) somewhat,(4) moderatley,(5) quite a bit),(6) a lot,(7) very much

These last questions ask what you know and feel about your anti-clot treatment.

**4a. How well do you feel that you understand the medical reason for your anti-clot treatment?**

1. not at all,(2) a little),(3) somewhat,(4) moderatley,(5) quite a bit),(6) a lot,(7) very much

**4b. How much do you feel reassured because of your anti-clot treatment?**

1. not at all,(2) a little),(3) somewhat,(4) moderatley,(5) quite a bit),(6) a lot,(7) very much

**4c. How much do you worry about your anti-clot treatment?**

1. not at all,(2) a little),(3) somewhat,(4) moderatley,(5) quite a bit),(6) a lot,(7) very much

**4d. How much do you worry about bleeding and bruising?**

1. not at all,(2) a little),(3) somewhat,(4) moderatley,(5) quite a bit),(6) a lot,(7) very much

**4e. How much do you worry about the bad things (for example, stroke) which your anti-clot treatment is intended to prevent?**

1. not at all,(2) a little),(3) somewhat,(4) moderatley,(5) quite a bit),(6) a lot,(7) very much

**4f. Overall, how much has anti-clot treatment had a positive impact on your life?**

1. not at all,(2) a little),(3) somewhat,(4) moderatley,(5) quite a bit),(6) a lot,(7) very much

**4g. Overall, how much has anti-clot treatment had a negative impact on your life?**

1. not at all,(2) a little),(3) somewhat,(4) moderatley,(5) quite a bit),(6) a lot,(7) very much

**4h. Overall, how satisfied are you with your anti-clot treatment?**

1. not at all,(2) a little),(3) somewhat,(4) moderatley,(5) quite a bit),(6) a lot,(7) very much

**4i. Compared with other treatments you have had, how difficult is your anti-clot treatment to manage?**

1. not at all,(2) a little),(3) somewhat,(4) moderatley,(5) quite a bit),(6) a lot,(7) very much

**4j. How likely would you be to recommend this form of anti-clot treatment to someone else with your disease or medical condition?**

(1) not at all,(2) a little),(3) somewhat,(4) moderatley,(5) quite a bit),(6) a lot,(7) very much
